# Cardiovascular Effects of Snake Toxins: Cardiotoxicity and Cardioprotection

**DOI:** 10.32607/actanaturae.11375

**Published:** 2021

**Authors:** A. S. Averin, Yu. N. Utkin

**Affiliations:** Institute of Cell Biophysics of the Russian Academy of Sciences PSCBR RAS, Pushchino, Moscow region, 142290 Russia; Shemyakin-Ovchinnikov Institute of Bioorganic Chemistry, Russian Academy of Sciences, Moscow, 117997 Russia

**Keywords:** bradykinin-potentiating peptides, snake venom, cardioprotector, cardiotoxin, natriuretic peptide, cardiovascular system

## Abstract

Snake venoms, as complex mixtures of peptides and proteins, affect various vital systems of the organism. One of the main targets of the toxic components from snake venoms is the cardiovascular system. Venom proteins and peptides can act in different ways, exhibiting either cardiotoxic or cardioprotective effects. The principal classes of these compounds are cobra cardiotoxins, phospholipases A2, and natriuretic, as well as bradykinin-potentiating peptides. There is another group of proteins capable of enhancing angiogenesis, which include, e.g., vascular endothelial growth factors possessing hypotensive and cardioprotective activities. Venom proteins and peptides exhibiting cardiotropic and vasoactive effects are promising candidates for the design of new drugs capable of preventing or constricting the development of pathological processes in cardiovascular diseases, which are currently the leading cause of death worldwide. For example, a bradykinin-potentiating peptide from *Bothrops jararaca *snake venom was the first snake venom compound used to create the widely used antihypertensive drugs captopril and enalapril. In this paper, we review the current state of research on snake venom components affecting the cardiovascular system and analyse the mechanisms of physiological action of these toxins and the prospects for their medical application.

## INTRODUCTION

Cardiovascular diseases (CVDs) are a vast group of heart and blood vessel diseases of various etiologies. They lead to impairment of the normal functions of various organs and, in severe cases, death. They put a huge burden on health care systems and the economy around the world. According to WHO estimates, more than 17 million people die from heart diseases every year. By 2030, this number is estimated to exceed 23 million. The leading causes of death are strokes and coronary heart diseases, which account for 31% of all deaths. In Russia, this indicator stands at 57%. Currently, a large number of drugs with various mechanisms of action exist, and they are used for the treatment of CVDs. Naturally, all possess certain side effects. For example, antiplatelet agents and anticoagulants can cause gastrointestinal tract complications and intracranial bleeding. The most common side effects of angiotensin-converting enzyme inhibitors are arterial hypotension, paroxysmal unproductive dry cough, angioedema of the upper respiratory tract, cholestasis, hyperkalemia, proteinuria, and impaired renal function. The use of β-blockers can be accompanied by a number of side effects, both cardiac (weakening of the pumping function of the heart, bradycardia, etc.) and extracardiac (drowsiness, depression, bronchospasm, etc.). In addition, a significant problem is associated with the insufficient efficacy of drug therapy for a number of CVDs, something that is especially pronounced in patients with concomitant pathologies. For example, a serious challenge in modern medicine is the chronic heart failure that is increasingly common in many cases and is difficult to correct. All these obstacles speak to the need for more effective drugs, with a fundamentally new mechanism of action: drugs that are free of the limitations typical of existing medicines.

**Table:** Snake venom toxins that affect the CVS

Toxin	Molecular weight, kDa	Main biological target	Effect on CVS
Bradykininpotentiating peptides	1.5–2.0	Angiotensin-converting enzyme	Lowering of blood pressure through a decrease in the concentration of angiotensin II and an increase in the concentration of bradykinin [3]
Natriuretic peptides	2.5–5.5	Natriuretic peptide receptors A, B, and C	Lowering of blood pressure through a reduction in vascular resistance (due to a decrease in the influx of calcium ions into muscle cells) and a decrease in the volume of circulating blood (due to an increase in the volume of excreted urine) [4, 5, 6]
Sarafotoxins	2.3–2.7	Endothelin type A (ETA) and B (ETB) receptors	Increased vasoconstriction followed by narrowing of the bronchi and increased airway resistance as well as an increase in hydrostatic pressure of microvessels in the lungs, which leads to their edema. Failure of various parts of the heart, mainly the left ventricle [7, 8]
Three-finger toxins	6.2–8.0	Cell membranes, adrenergic receptors, cholinergic receptors	Suppression of contractility and irreversible contracture of the myocardium; lowering blood pressure; cardioprotection [9, 10, 11]
Cysteine-rich secretory proteins (CRISPs)	23–25	Voltage-gated ion channels	Inhibition or activation of aortic smooth muscle contraction [12, 13]
Alternagin-C	21.7	Integrin α2β1 and VEGFR-2	Enhancement of cardiac activity; protection against hypoxia/reoxygenation-induced cardiomyocyte negative inotropism [14, 15]
Endothelial vascular growth factors	24–26	Receptor tyrosine kinases VEGFR-1, VEGFR-2, and VEGFR-3	Cardioprotective effect; reduction in reperfusion injury to the heart and infarct size [16, 17]
Phospholipases A2	13–14	Cell membrane, secretory PLA2 receptors	Cardiotoxicity; myocardial contracture, vascular relaxation [18, 19, 20, 21]

## SNAKE VENOMS: COMPOSITION AND PROPERTIES

Snake venoms are complex mixtures of compounds with high biological activity and a high selectivity of action. These compounds are capable of affecting various systems of the organism, but their main targets are the nervous and cardiovascular systems (CVS). Depending on the most affected system, snake venoms are classified as neurotoxic and hemotoxic. Neurotoxic venoms are typical of snakes from the Elapidae family (cobras, kraits, mambas, coral snakes, and some other snakes) and contain mainly non-enzymatic toxins that block nerve impulse conduction. Hemotoxic venoms are typical of snakes from the Viperidae family (vipers, moccasins, rattlesnakes and some other snakes). Hemotoxic venoms consist mainly of enzymes that cause coagulopathy. Both types of venoms can contain toxins that affect the CVS, with the venom of one individual comprising up to several hundred different peptides and proteins. Snake venom proteins and peptides affecting the CVS can act in different ways, causing both cardiotoxic and cardioprotective effects. These compounds belong to different toxin families and interact with various biological targets in the organism. Snake venom poisoning is associated with a number of cardiovascular effects, including hypotension, myocardial infarction, cardiac arrest, hypertension, brady- or tachycardia, and atrial fibrillation [1]. Given the multiplicity of the effects, it may be stated that snake venom is a rich source of compounds that affect the CVS. These compounds, with various biological activities, could be of significant pharmacological value and represent a promising basis for the development of new drugs.

It should be noted that snake venoms contain a large number of peptides and proteins that affect blood cells and enzyme systems. However, in this review, we will limit ourselves to the consideration of toxins that directly affect the CVS.

**Snake toxins affecting the CVS**

As has already been noted, snake venoms contain a number of compounds that affect the CVS. By their chemical nature, these can be low-molecular-weight organic compounds (e.g., adenosine), peptides, and proteins. These snake venom components include, in particular, bradykinin-potentiating peptides (BPPs), natriuretic peptides (NPs), sarafotoxins (SRTXs), and three-finger toxins (TFTs), including cobra cardiotoxins (CTs), phospholipases A2 (PLA2s), and vascular endothelial growth factors (VEGFs) [2] (*Table*). These toxins affect the heart muscle, vascular smooth muscles, and the capillary vascular bed.

**Peptide toxins**

**Fig. 1:**
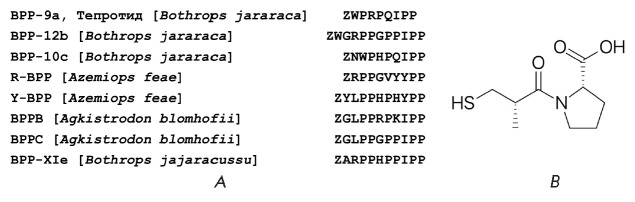
Amino acid sequences of BPPs (A) and the structure of captopril (B). Z is a pyroglutamic acid residue

*Bradykinin-potentiating peptides (BPPs). *BPPs consist of 5–14 amino acid residues and contain a proline-rich region [2, 22] (*Fig. 1A*). In the organism, BPPs inhibit the angiotensin-converting enzyme (ACE) that breaks down angiotensin I, converting it into angiotensin II, a potent vasoconstrictive and hypertensive agent. BPPs lower blood pressure by blocking the formation of angiotensin II. In addition, ACE is also capable of cleaving bradykinin that possesses hypotensive activity and inhibition of the enzyme enhances the effect of bradykinin and leads to vasodilation and decreased cardiac output [3]. The first antihypertensive drug of its class, the ACE inhibitor captopril (*Fig. 1B*), was derived from a BPP (teprotide) from the venom of the snake *Bothrops jararaca*.

It should be noted that ACE is a two-domain enzyme. The generation of a potent vasoconstrictor, angiotensin II, occurs primarily through the action of the ACE C-domain. Both homologous domains hydrolyze bradykinin, with the C-domain being somewhat more efficient [23]. The ACE inhibitors including captopril commonly used in clinic are not domain-selective. However, they can lead to life-threatening angioedema associated with the systemic accumulation of bradykinin upon the inhibition of both ACE domains. Therefore, the development of a domain-specific inhibitor is urgently needed. Selectivity of action on a certain domain was found for some BPPs. For example, the decapeptide Bj-BPP-10c (*Fig. 1*) is 400-fold more selective for the active site in the C domain (Ki = 0.5 nM) than for the N domain (Ki = 200 nM) [24]. The opposite was discovered for Bj-BPP-12b (*Fig. 1*), which is more selective for the N domain (Ki = 5 nM) and 30-fold less effective for the C domain [25]. The BPPs R-BPP and Y-BPP, which we uncovered in the venom of the viper *Azemiops feae* (*Fig. 1*) [26], are more similar to peptides exhibiting specificity for the ACE C domain and may be considered as a basis for the development of C domain-selective drugs, which would differ structurally from captopril.

In addition to inhibiting ACE, some BPPs kinetically modulate the activity of argininosuccinate synthase* in vitro *and *in vivo*, which ultimately leads to the production of nitric oxide (NO) in endothelial cells and a decrease in blood pressure [27]. Modulation of argininosuccinate synthase not only stimulates the production of nitric oxide, but also enhances the synthesis of protective molecules, such as polyamines (spermine, spermidine, and putrescine) and agmatine, which, as was shown in one of our studies, can lead to a positive inotropic effect even upon reduced activity of Ca^2+^-ATPase of the sarcoplasmic reticulum [28], a characteristic of heart failure [29]. Recently, one of the mentioned BPPs was shown to protect SH-SY5Y neuroblastoma cells from the oxidative stress caused by hydrogen peroxide [30]. It should be noted that post-heart-attack reperfusion induces oxidative stress, leading to severe cardiac dysfunction. Therefore, biologically active compounds that reduce oxidative stress can be considered a promising therapeutic strategy for heart diseases. Potentially, BPPs could be such compounds. In addition, BPPs have a direct effect on the components of the cardiovascular system. For example, in some cases there is no correlation between ACE inhibition and the hypotensive effect [31], and a BPP from the venom of the cobra *Naja haje haje* dose-dependently reduces the contractility of the rat atria [32]. The BPP Bj-PRO-5a was also found to cause vasodilation by interacting with the muscarinic cholinergic receptors M1 and bradykinin receptors BK_B2_ and triggering NO synthesis by the endothelium [33]. There is evidence that BPPs can enhance the effect of bradykinin by increasing the sensitivity of its receptors. But the mechanism of this action has not been elucidated [34]. Therefore, many physiological mechanisms, both central and peripheral, underlie the general hypotensive effect of BPPs.

**Fig. 2:**
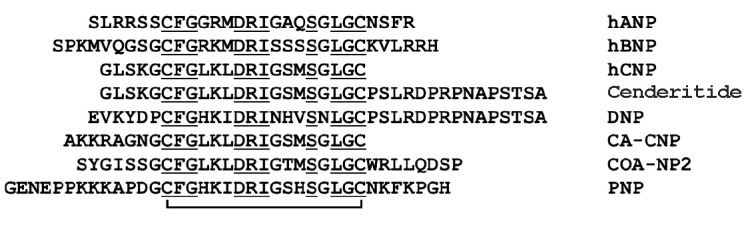
Amino acid sequences of NPs. Identical amino acid residues are underlined. The disulfide bond is shown as a line connecting cysteine residues. hANP and hBNP are human atrial and brain NPs, respectively. hCNP is the human C-type NP. DNP is an NP from *Dendroaspis angusticeps *mamba venom (UniProtKB -P28374), CA-CNP is a C-type NP from *Crotalus atrox *venom (P0CV87), COA-NP2 is an NP from *C. oreganus abyssus *venom (B3EWY2), and PNP is an NP from* Pseudocerastes persicus *venom (P82972)

*Natriuretic peptides. *A number of snake venom peptides mimic the actions of endogenous peptides. These compounds include, in particular, natriuretic peptides (NPs). NPs contain about 20 to 50 amino acid residues and are based on a conserved 17-aa sequence confined by a disulfide bond (*Fig. 2*). There are three isoforms of mammalian NPs: namely atrial NP (ANP), brain NP (BNP), and C-type NP (CNP). NPs also include urodilatin, which is an extended ANP derived from a precursor using an alternative processing system. In addition, a D-type NP (DNP) and ventricular NPs (VNPs) are sometimes distinguished. The DNP is a unique NP isolated only from the venom of the eastern green mamba* Dendroaspis angusticeps*. To date, VNP expression has been confirmed only in the heart of primitive bony fish [35]. Atrial NPs are the key hormones in the regulation of pressure–volume homeostasis. These peptides interact with membrane-bound NP receptors (NPRs) in the heart, vasculature, and kidneys, reducing blood pressure and circulation volume. The effects of NPs can be quite diverse: in mice, endogenous BNPs and CNPs increase the heart rate [36], while in the rat myocardium, CNP causes a decrease in contractility [37]. A common property of NPs is the ability to induce an increase in NO production and activate protein kinase G, which mediates their vasorelaxant effect [4, 38] in most cases; however, some NPs can also induce relaxation on endothelium-denuded aortic preparations [38, 39]. Therefore, NPs cause a whole spectrum of physiological effects that can potentially be used to correct CVD. For example, intravenous infusion of NPs improves the hemodynamic status in patients with heart failure, but sometimes it is accompanied by severe hypotension, which requires the development of NP analogs lacking these side effects.

NPs are found in the venoms of various snake species, including the eastern green mamba *D. angusticeps* [40], rattlesnakes *C. atrox *and *C. oreganus abyssus* [4], and others [41, 42] (*Fig. 2*). Their action leads to vascular relaxation and a decrease in myocardial contractility [4, 6]. Venom NPs are of interest as a basis for the creation of NPs with a longer half-life and improved selectivity for vessels and kidneys [43]. In this regard, snake venom NPs are considered a good basis for the design of NPs with therapeutic potential. To date, venom NPs have been used to develop several analogs with the prospect of clinical application; of these, the most successful agent is cenderitide [5]. Cenderitide is a chimeric peptide consisting of a human C-type NP fused to the C-terminal fragment of an NP from the venom of the eastern green mamba* D. angusticeps* (*Fig. 2*). Cenderitide was developed to co-activate two NP receptors, in particular the guanylyl cyclases pGC-A and pGC-B, for improving renal function, but without clinically significant hypotension. Cenderitide was shown to be well tolerated by healthy volunteers, without side effects and to activate cGMP, which corresponded to the activation of the NP receptor. Cenderitide induced a minimal decrease in blood pressure, along with natriuresis and diuresis. Preliminary experiments in patients with heart failure demonstrated good tolerance and no side effects. Cenderitide is a promising agent for the treatment of heart failure.

**Fig. 3:**
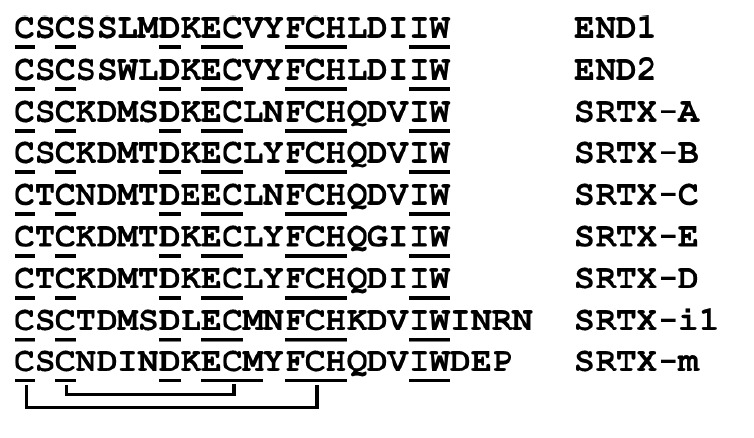
Amino acid sequences of endothelins and sarafotoxins. Disulfide bonds are shown as lines connecting cysteine residues. END1 (UniProtKB – P05305) and END2 (P20800) are human endothelin 1 and 2, respectively. SRTX-A (UniProtKB – P13208), SRTX-B (P13208), SRTX-C (P13208), SRTX-E (P13208), and SRTX-D (P13211) are sarafotoxins A, B, C, E, and D from *A. engaddensis *venom, respectively. SRTX-i1 (P0DJK0) is sarafotoxin i1 from* A. irregularis *venom; SRTX-m (Q6RY98) is sarafotoxin m from the venom of *A. microlepidota microlepidota*

*Sarafotoxins. *Sarafotoxins (SRTXs), which possess strong vasoconstrictive properties, are short peptide toxins found in the venom of snakes of the genus* Atractaspis*. These peptides, which have a high degree of identity with endothelins, recognize and bind endothelin receptors. SRTXs from the venom of* Atractaspis engaddensis *contain 21 amino acid residues and two disulfide bonds (*Fig. 3*); the toxins of other snake species have an extended C-terminal fragment. They stimulate endothelin receptors and increase vasoconstriction, followed by left ventricular dysfunction, bronchospasm, and increased airway resistance. SRTX-B binds to endothelin receptors with high affinity and causes cardiac arrest and death in mice within minutes of intravenous administration.

The contractile response of vessels to sarafotoxins is mainly associated with the input of extracellular calcium through L-type calcium channels, while intracellular calcium stores released through ryanodine and IP-3 channels play a relatively small role [8].

The effect of SRTX-C can be multidirectional. For example, a small negative inotropic effect is observed in the intact right papillary muscles of a rabbit, while a strong increase in contractility occurs upon removal of the endothelium and inhibition of nitric oxide or prostaglandin signaling [44]. In the human myocardium, SRTX-C causes an increase in contractility associated with arrhythmia, which is most pronounced in the right atrium compared with other myocardial tissues [45]. Intracoronary administration of SRTX-C is known to lead to a decrease in cardiac output and an increase in the time parameters of cardiac contraction in pigs [46]. In this case, the classic short SRTXs from* A. engaddensis *cause disturbances in the left ventricle, while SRTX-m from the venom of *A. microlepidota microlepidota *[47] leads to a dysfunction of the right ventricle [7].

In scientific research, SRTXs are used to label endothelin receptors and develop vasospasm models [48].

**Non-enzymatic protein toxins**

*Three-finger toxins. *Three-finger toxins (TFTs) constitute one of the most abundant families of snake venom toxins. TFTs consist of 57–82 amino acid residues; structurally, TFT molecules are represented by three β-structural loops extending from a compact hydrophobic core that is stabilized by four conserved disulfide bonds. The biological properties of TFTs are very diverse; a number of TFTs affect the CVS [11].

**Fig. 4:**
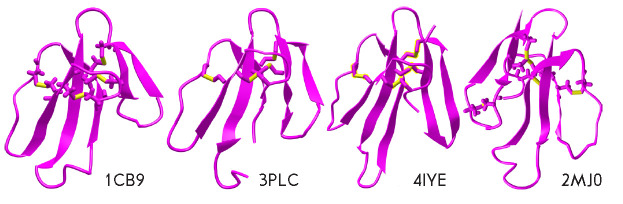
Spatial structures of some three-finger toxins. Cardiotoxin II from *Naja oxiana *(PDB code – 1CB9), β-cardiotoxin from *Ophiophagus hannah *(3PLC), toxin ρ-Da1a from *Dendroaspis angusticeps *(4IYE), and weak toxin WTX from *Naja kaouthia *(2MJ0). The structures of cardiotoxin II and WTX were established by NMR, the structures of β-cardiotoxin and toxin ρ-Da1a were determined by X-ray analysis. Disulfide bonds are highlighted in yellow

Cytotoxins, also called cardiotoxins (CTs), are TFTs that consist of about 60 amino acid residues and contain four disulfide bonds (*Fig. 4*). A common property of cytotoxins is their direct interaction with the membrane to form an ionic pore, which causes depolarization of a cell and its death. This is most clearly seen in the heart, which imparts to this group the alternative name cardiotoxins. Despite the fact that the amino acid sequences of CTs are very similar [49], their biological activity can differ significantly [50, 51]. Most studies have shown that CTs begin to act even at a concentration of less than 1 μM, initially causing an increase in contraction, followed by a decrease and a concomitant rise in the resting tension [9, 52, 53]. Comparison of various myocardial tissues showed that the effect of CTs on the ventricular tissue is more pronounced than that on the atria [54, 55]. Usually, contracture caused by the CT effect is irreversible and leads to cell death [10, 53, 56, 57, 58]. Initial cell depolarization results in an increase in the intracellular calcium concentration from intraand extracellular sources [53]. The role of individual calcium-transporting mechanisms in the development of CT effects can vary depending on myocardial characteristics. For example, the L-type Ca^2+^ current in neonatal rat cardiomyocytes is the leading mechanism for increasing the level of intracellular calcium [57], while the blocking of this mechanism in adult cardiomyocytes [53] and guinea pig myocardium [10] does not prevent the development of contracture. It should be noted that the CT effect depends on the concentration of extracellular calcium, high concentrations of which (about 10 mM) block CT effects [10, 53, 57]. CTs induce a long-term increase in the intracellular calcium concentration, accompanied by the activation of peptidases inside the cell and disintegration of the cardiomyocyte structure [53, 57], which results in a chain of pathological processes leading to cell death [53] through the necrotic mechanism [58].

In blood vessels, as in other muscle tissue types, CTs cause contracture; in this case, a transient relaxation effect caused by the activation of endothelial cells is observed in phenylephrine-precontracted aortic rings [59]. The contractile response involves both the input of extracellular calcium [59] and its release from intracellular stores [60]. The effects of CT on both the smooth muscle and endothelial cells are curbed by high calcium concentrations [61, 62].

Despite the fact that CTs are very toxic compounds highly unlikely to exert a positive effect on the heart and blood vessels, CTs (fraction 1 from *N. naja siamensis*) have been reported to induce a positive inotropic response with no contractures at a dose of up to 100 μg/mL [10], which may be useful in myocardial pathology, accompanied by a decrease in the pumping function of the heart. Currently, only cardiac glycosides are used as drugs with a positive inotropic effect, which, according to the DIG study, is particularly good on patients with chronic heart failure with a reduced left ventricular ejection function [63]. Therefore, searching for new compounds possessing a cardiotonic effect remains a priority. However, there is nary information about studies of CTs with such a profile of action in pathological myocardium models; e.g., in SHR rats with reduced cardiac contractility. CTs may also be useful in exploring the mechanisms of dystrophic vascular calcification [64]. In this case, CTs are used as a methodological approach for triggering a cascade of pathological events that may be used to investigate vasoprotective mechanisms.

The TFT group also includes venomous cardiotoxin- like proteins [65] that interact with the different adrenergic receptors (ARs) abundant in the cardiovascular system. For example, a number of toxins have been isolated from the venom of the eastern green mamba *D. angusticeps*. These specifically interact with different subtypes of adrenergic receptors: ρ-Da1a (*Fig. 4*) selectively blocks the α1A-AR subtype [66, 67], and ρ-Da1b blocks all three α2-AR subtypes [68, 69]. The so-called muscarinic toxins MT1 and MT2 reversibly bind to α1-ARs [70]. The toxins MTβ and CM-3, similar to ρ-Da1a, were isolated from the venom of the black mamba *D. polylepis*; however, they interact with higher affinity with the α1B- and α1D-AR subtypes [71].

β-Cardiotoxin was isolated from the venom of the king cobra *Ophiophagus hannah* (*Fig. 4*). It is capable of blocking β1 and β2 ARs [72]. This leads to a decrease in the heart rate *in vivo *and *in vitro *without noticeable cytotoxicity, which may be associated with the inability of β-cardiotoxin to directly interact with the membrane, due to some of its structural features [73]. Later, a cytotoxic effect on cultured smooth muscle cells and no effect on skeletal cells and cardiac myocytes were shown in [74]. Interestingly, the study revealed direct negative inotropic and lusitropic effects, with the intracellular calcium concentration in systole remaining unchanged. These data may indicate the existence of direct mechanisms of β-cardiotoxin action which are not associated with AR activation, and the ability of ARs to alter the sensitivity of myofilaments to calcium ions. The presence of compounds in the TFT group which interact highly specifically with individual AR subtypes may be of great utility in pharmacological studies, because each of the three subtypes plays an important role in CVS pathologies and their correction. For example, blocking β-ARs is one of the main directions in the therapy of various forms of hypertension and chronic heart failure [75, 76]; activation of α1-ARs may be considered as a compensatory pathway in the desensitization of the β-AR pathway [77, 78, 79]; and α2 activation may be considered as a cardioprotective pathway preventing adrenergic overload of the heart [80, 81] and, as shown in our publications, blocking the development of arrhythmias and Ca-overload in cardiomyocytes [82, 83].

One of the TFT groups is composed of the so-called non-conventional toxins that contain an additional disulfide bond in the N-terminal fragment and are usually characterized by low toxicity. Interestingly, one of the representatives of this group, toxin WTX, when administered intravenously, reduced blood pressure in rats [84] by affecting cholinergic transmission.

Another TFT group is represented by toxins affecting the activity of various ion channels and the receptors present in the CVS. However, since there are no data on the effect of these toxins on the CVS, they are not discussed in this review.

*Other types of toxins. *There are a number of other toxins that affect the CVS and lack enzymatic activity. These include toxins of the CRISP (Cysteine-RIch Secretory Protein) family, which are 23–25 kDa proteins containing eight disulfide bonds. For example, ablomin from the venom of *A. blomhoffi *and some similar toxins blocked the contraction of rat arterial smooth muscles caused by a high concentration of potassium ions. Ablomin is supposed to inhibit the voltage-gated influx of extracellular calcium, which causes vascular contraction [13]. Natrin of *N. atra *venom induces a contractile response in the endothelium-denuded thoracic aorta of mice [85]. Further experiments showed that natrin is able to block the high-conductance calcium- activated potassium channels (BKCa) that play a significant role in the regulation of the vascular tone. In addition, natrin can block the skeletal isoform of the ryanodine receptor [86] and voltage-gated potassium channels KV1.3 [87].

The protein alternagin-C, isolated from *Bothrops alternatus *snake venom, has a very interesting effect on the CVS [88]. This protein can induce the expression of the vascular endothelial growth factor, proliferation and migration of endothelial cells, enhance angiogenesis, and increase the viability of myoblasts. Therefore, this peptide can play a crucial role in the mechanisms of tissue regeneration. A study of the alternagin-C effect on the cardiac function *in vitro *in freshwater fish showed that the protein enhances cardiac activity, promoting a significant increase in the contraction force and the rate of contraction and relaxation with a concomitant decrease in time to peak tension and improving the cardiac pumping capacity [14]. Alternagin-C improves the cardiac function by increasing the efficiency of calcium ion transport, which leads to positive inotropism and chronotropism [14]. Therefore, this protein can improve the regulation of the cardiac output, which indicates the possibility of its use in the treatment of cardiac contractile dysfunction. Also, the effect of alternagin-C on hypoxia/reoxygenation in isolated ventricular strips of fish and on morphological changes and the density of blood vessels was studied [15]. Treatment with alternagin-C provided protection of cardiomyocytes from the negative inotropism caused by hypoxia/reoxygenation. This protein also stimulated angiogenesis and improved excitation–contraction coupling during hypoxic conditions. These results indicate a new therapeutic strategy for the treatment of diseases associated with ischemia.

A number of snake venom proteins mimic the effects of the endogenous factors that regulate the physiological functions of the body. Regarding the CVS, of interest is a group of proteins, such as vascular endothelial growth factors (VEGFs), that can enhance angiogenesis and increase vascular permeability. VEGFs exhibit hypotensive [17] and cardioprotective effects [16]. Three receptor tyrosine kinases, known as VEGFR-1, VEGFR-2, and VEGFR-3, act as VEGF receptors. VEGFR-1 and VEGFR-2 are present primarily on vascular endothelial cells and mediate several major angiogenic activities: for example, endothelial cell proliferation. Reperfusion injury of the heart includes, among various mechanisms, coronary endothelial dysfunction. VEGF activates endothelial cells and has a cardioprotective effect. Snake venoms contain proteins that induce VEGF-like effects in endothelial cells. A number of the proteins that interact with VEGF receptors have been isolated and characterized [16]. In this case, some snake proteins selectively interacted with VEGFR-2, e.g., vammin from *V. ammodytes*, while others exhibited selectivity for VEGFR-1, e.g., VEGF from *T. flavoviridis *[89]. It was found that a protein from *V. lebetina*, like VEGF, significantly reduces reperfusion injury and infarct size thanks to a stimulation of VEGFR-2 receptors [16]. However, its activity proved somewhat less impactful than that of VEGF. Probably, snake venoms contain proteins with the same cardioprotective activity as in VEGFs, but without their inherent side effects.

**Enzymatic protein toxins**

Of the many enzymes present in snake venoms, so far only phospholipases A2 (PLA2s) have exhibited direct action on the CVS. Snake venom PLA2s belong to the class of secreted lipolytic enzymes that hydrolyze the ester bond of glycerophospholipids at the Sn2 position to form lysophospholipids and free fatty acids [90], which serve as a source for the synthesis of the secondary mediators involved in the physiological processes taking place in cells. However, the effect of lipolysis products is not decisive for cardiotoxicity [91]; rather, damage to the cell membrane plays a leading role here [92]. In addition, some of the physiological effects are mediated through interaction with secretory PLA2 receptors [93]. Snake venom PLA2s can lower blood pressure through the production of arachidonic acid, a precursor of cyclooxygenase metabolites (prostaglandins or prostacyclins). It should be noted that systemic administration of high PLA2 doses can cause disruptions in the structure of myocardial tissue [21, 94] and its functioning, such as bradycardia and atrioventricular block [95, 96]. Interestingly, some of the cardiotoxic effects observed in *in vivo *animal studies are due to disruptions in the composition of the internal medium of the organism [97, 98]. PLA2s derived from the venoms of different snakes can differ significantly in their cardiotoxicity; e.g., PLA2s from *O. hannah *and* N. nigricollis *cause intracellular structural changes and contracture [94, 96, 99], in contrast to the PLA2 from the venom of *N. naja atra *that lacks cardiotoxicity [99]. The inotropic effect can be multidirectional; usually, contractility decreases after short growth, accompanied by an increase in the resting tension that can be transformed into contracture [20, 21, 99]. Acting on blood vessels, PLA2s usually exert a vasorelaxant effect that is independent of the endothelium and is partially mediated by an increase in cGMP in smooth muscle cells [18, 19]. The PLA2 effects can be significantly weakened by suramin [100] and a phospholipase A2 inhibitor: *p*-bromophenacyl bromide [21, 97]. As in the case of CTs, the PLA2 effects can be blocked by a high concentration of calcium ions, while calcium channel blockers are ineffective [19, 96]. PLA2s and CTs induce myocardial contracture, whereas PLA2 induces vascular relaxation.

## PROSPECTS OF SNAKE VENOMS IN DRUG DEVELOPMENT AND POSSIBLE ROADBLOCKS

Snake venom toxins highly efficiently and selectively affect the various systems in living organisms, including the CVS, which makes them very attractive as a basis for drug design. The main disadvantages of toxins are their high toxicity and irreversibility of action; i.e., the inability of an affected system to return to its original state. Given the abovementioned data, there are many highly active cardiotropic or vasoactive snake toxins which may be used in the future as a basis for the development of new drugs. Some of these proteins and peptides have demonstrated that they can be highly selective tools in research into physiological processes. Others have been used as probes for potential therapeutic targets or a basis for the development of therapeutic agents.

We have already considered the antihypertensive drug captopril (*Fig. 1*) derived from a bradykininpotentiating peptide of the South American jararaca. Another drug based on this peptide is enalapril, (S)- 1-[N-[1-(ethoxycarbonyl)-3-phenylpropyl] -L-alanyl]- L-proline, that is currently widely used in hypertension.

A promising drug is cenderitide, produced by the addition of a 15 aa C-terminal fragment of a natriuretic peptide isolated from *D. angusticeps *venom to the fulllength human C-type natriuretic peptide. It may be used in heart failure. Cenderitide has already passed the first and second phases of clinical trials, albeit with a small number of participants, and has shown promise in maintaining left-ventricular function in myocardial infarction.

There are good prospects for alternagin-C, its analogs, and endothelial vascular growth factor analogs from snake venoms for the development of drugs that prevent reperfusion injuries. However, it remains necessary to evaluate the *in vivo *activity of these proteins and their stability in the organism. To date, there are still no data on clinical studies of these proteins.

In conclusion, it should be noted that, despite their existing drawbacks, a number of snake venom peptides and proteins that affect the CVS have good prospects as a basis for the development of new drugs.

## Abbreviations

ACE: angiotensin-converting enzyme
AR: adrenergic receptor
BPP: bradykinin-potentiating peptide
CT: cardiotoxin
NP: natriuretic peptide
NPR: NP receptor
CVD: cardiovascular disease
CVS: cardiovascular system
TFT: three-finger toxin
PLA2: phospholipase A2
VEGF: vascular endothelial growth factor
ANP: atrial NP
BNP: brain NP
CNP: C-type NP
SRTX: sarafotoxin
VEGFR: vascular endothelial growth factor receptor.
